# Long-term outcome after mitral valve replacement using biological versus mechanical valves

**DOI:** 10.1186/s13019-019-0943-6

**Published:** 2019-06-28

**Authors:** Ayse Cetinkaya, Julia Poggenpohl, Karin Bramlage, Stefan Hein, Mirko Doss, Peter Bramlage, Markus Schönburg, Manfred Richter

**Affiliations:** 1 Department of Cardiac Surgery, Kerckhoff-Heart Center Bad Nauheim, 61231 Bad Nauheim, Germany; 2 Institute for Pharmacology and Preventive Medicine, Bahnhofstraße 20, 49661 Cloppenburg, Germany

**Keywords:** Mitral valve replacement, Biological valve, Mechanical valve

## Abstract

**Background:**

This study compared long-term outcomes of biological and mechanical mitral valve replacement (MVR) in patients requiring replacement of the mitral valve where repair was not feasible.

**Methods:**

A single-centre registry of patients receiving MVR between 2005 and 2015 was established. Thirty-day mortality and long-term outcomes were analysed and compared.

**Results:**

Three hundred twenty four patients underwent MVR (265 biological; 59 mechanical valves). Patients receiving biological valves were older (*p* < 0.001), had a higher log EuroSCORE (*p* < 0.001) and received less minimally invasive surgery (*p* < 0.001).

Immediate procedural mortality was 1.9%, which only occurred in the biological valve group. At 30 days, 9.0% of patients had died, 4.0% experienced stroke, 8.0% received a pacemaker and 10.5% suffered an acute renal failure. The rate of re-thoracotomy (14.2%) was lower in the biological (12.5%) than in the mechanical valve group (22.0%; adjOR 0.45 [0.20–1.00]; *p* = 0.050). Frequent long-term complications were stroke (9.2%) and bleeding (4.8%), with bleeding complications being higher in the mechanical valve group (*p* = 0.009). During the follow-up period biological valves showed a numerically higher survival rate during the first years, which shifted after 3 years in favour of mechanical valves. At 10 years, survival rates were 62.4% vs. 77.1% in the biological and mechanical valve groups (*p* = 0.769). Hazard ratio after adjustment was 0.833 (95% CI 0.430–1.615).

**Conclusion:**

These data confirm that mechanical valve implantation is associated with an increased risk of bleeding. While there was a potential survival benefit during the first years after surgery for patients receiving a biological valves the difference became insignificant after a follow-up of 10 years.

**Electronic supplementary material:**

The online version of this article (10.1186/s13019-019-0943-6) contains supplementary material, which is available to authorized users.

## Background

In accordance with guidelines from the European Society of Cardiology (ESC) and the European Association for Cardio-Thoracic Surgery (EACTS), mitral valve replacement (MVR) is a surgical procedure that is used when the patient’s heart valve is so severely compromised that mitral valve (MV) repair is no longer a viable option [1, 2]. The American Heart Association (AHA) guidelines recommend MV surgery for asymptomatic patients with chronic severe primary mitral regurgitation (MR) and left ventricular (LV) dysfunction [3, 4]. Again, MV repair is preferred over MVR when possible [3, 4].

Data on the choice of biological versus mechanical MV is controversial. Biological MVs are generally considered to be associated with lower bleeding complications due to the need for less or no anticoagulation, but they lack of durability. Mechanical valves are durable, but are associated with thromboembolism and bleeding complications mostly because of inevitable lifelong anticoagulation therapy [2, 5, 6].

In a study of 279 patients undergoing MVR (154 biological and 125 mechanical valves), clinically satisfactory results were obtained in both groups. After 15 years, fewer patients in the mechanical valve group were free of bleeding events (92.5% vs. 100%) and patients in the biological valve group had a lower freedom from thromboembolism (72.2% vs. 93.5%), valve failure (22.0% vs. 87.0%) and cardiac events (16.5% vs. 47.2%). The study concluded that the use of mechanical valves was preferential and biological valves should be reserved for patients older than 65 years and only be used with concomitant anticoagulant therapy [5]. Conversely, the results of a 10-year follow-up study published in 2001 showed no significant clinical differences between patient groups receiving biological and mechanical valves [7]. These results confirm the findings of a study from the 1980s [8].

This paper aims to compare the outcomes after biological versus mechanical MVR in patients who were not suitable or ineligible for MV repair.

## Methods

This study is a single-centre registry analysis of MVR procedures performed at the Kerckhoff-Heart Center, in Bad Nauheim, Germany, between January 2005 and December 2015. It was approved by the site’s Ethical Committee and complied with the Declaration of Helsinki and its amendments. Given the use of anonymised data already collected as part of routine diagnosis and treatment, written informed consent was neither feasible nor required.

### Patient population

All patients undergoing MVR at our site within the specified time period (January 2005–December 2015) were included. The study also included patients receiving MVR combined with tricuspid valve repair and ablation therapy, patent foramen ovale (PFO) or atrial septal defect (ASD) closure.

Age, comorbidity and lifestyle were considered when recommending biological valves to patients. Patients with a contraindication for vitamin K antagonists (VKA), women with a desire to become pregnant, certain professions (e.g., pilots) and those with a likely lack of compliance were also considered for biological valves even if they were less than 65 years of age.

Patients with endocarditis had their infection carefully resected. Patients with severe calcification and some of those with endocarditis received neochordae implanted at the mitral annulus. Patients with mitral insufficiency without calcification and without endocarditis who received MVR after two failed attempts had their anterior mitral leaflet (AML) resected and the posterior leaflet (PML) preserved to protect the subvalvular apparatus.

Exclusion criteria were a simultaneous coronary artery bypass grafting (CABG) or an aortic valve replacement. Of note, all patients with biological valves routinely receive acetylsalicylic acid (ASA) 100 mg daily and patients with mechanical valves and/or atrial fibrillation received VKA.

### Data and outcomes

In patients who had undergone an MVR, we checked electronic medical records (inpatient and outpatient notes and the results of any diagnostic testing). Clinical variables of interest were patient age, sex, comorbidity, prior cardiological interventions, echocardiography (pre-operative transthoracic echocardiogram [TTE], perioperative transoesophageal echocardiogram, one-week post-operative and upon follow-up) and relevant medical/surgical history. Follow-up data collected at the patient’s last follow-up hospital or outpatient visit were valve-related complications and echocardiography parameters.

### Statistics

Data were analysed using descriptive statistics, with categorical variables presented as absolute values and frequencies (%) and normally distributed continuous variables as means with standard deviations (SDs). Non-normally distributed continuous variables are presented as the median and interquartile range (IQR with the borders for the first and third quartile). Comparisons between biological and mechanical valve groups were carried out using a Student’s T-test with Levine’s homogeneity of variance or the Mann-Whitney U-test for continuous variables, as appropriate, and a Fisher’s exact or Chi-square test for categorical variables. For outcome analyses odds ratios (OR) were calculated by logistic regression. Survival analysis was presented as Kaplan-Meier (KM) curve. Hazard ratios (HR) were calculated by Cox-regression and adjusted for major baseline variables. Freedom from major complications (embolism/stroke and bleeding) were displayed as KM curves. For patients without a documented time-point for the documented event (embolism/stroke or bleeding), the time to death or the time of the last follow-up visit was used and divided by two for the KM curves. In all cases, a two-tailed *P*-value of < 0.05 was considered statistically significant. All statistical tests were performed using IBM SPSS Statistics software version 24.0 (Armonk, NY. IBM Corporation).

## Results

**Fig. 1:**
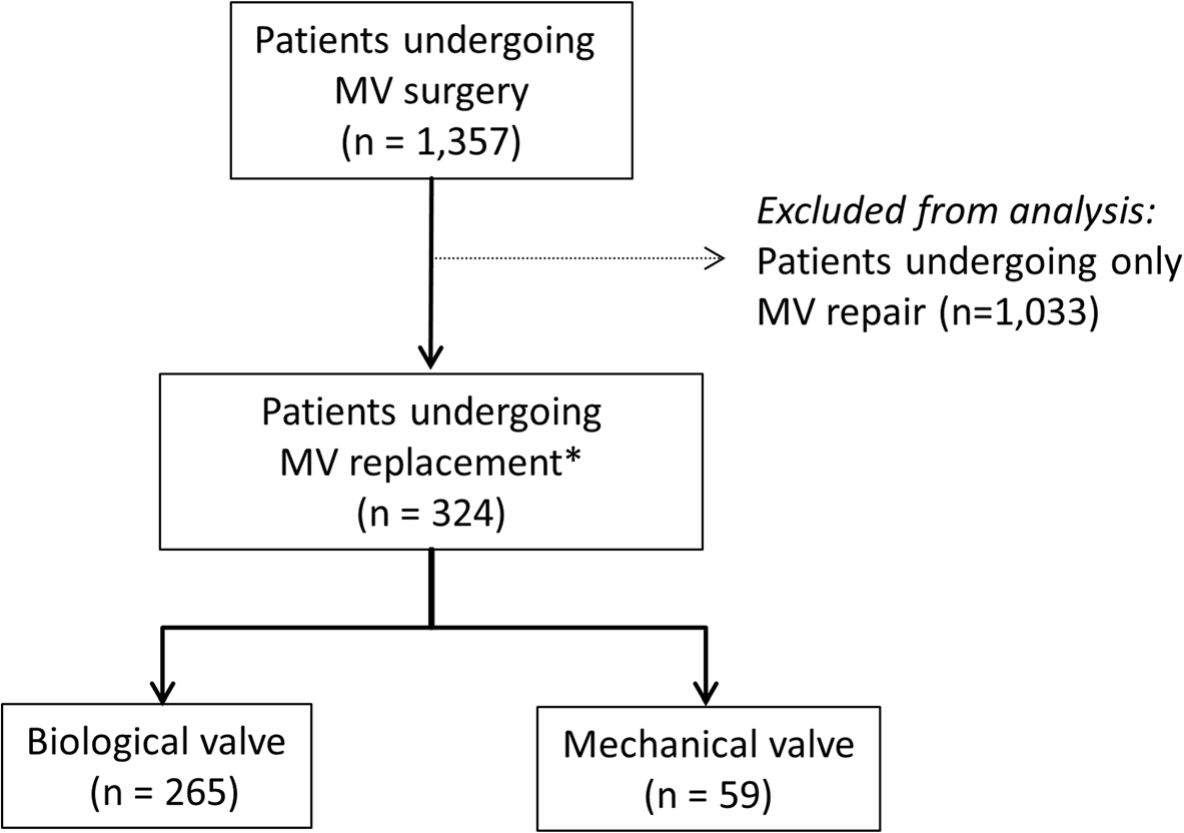
Flow Chart. *Legend:* MV, mitral valve. *123 patients underwent MV replacement after a MV repair was attempted

Between 2005 and 2015, 1357 patients received MV surgery at the Kerckhoff-Heart Center, including 324 patients receiving MVR. Of these patients, 265 patients received a biological valve and 59 a mechanical valve (Fig. 1).

### Patient characteristics

Patient characteristics, MV pathologies and echocardiographic parameters are presented in Tables 1 and 2. Overall, 49.7% of patients were female and 38.9% had atrial fibrillation. The majority of patients had degenerative MV disease (96.0%) with a dilated annulus (71.3%) and MV insufficiency grade ≥ II (96.6%). 28.7% of the patients presented with MV stenosis and 14.5% suffered from acute endocarditis. Patients receiving biological valves were older (71.0 vs. 56.0 years; *p* < 0.001) and had a significantly higher median log EuroSCORE I (8.4% vs. 4.5%; *p* < 0.001).

**Table 1:** Patient characteristics

	Total *N* = 324	Biological MVR *N* = 265	Mechanical MVR *N* = 59	*p*-value
Age in years	69.0 [58.0–76.0] [324]	71.0 [62.0–77.0] [265]	56.0 [50.0–62.0] [59]	< 0.001
≤ 65 years, %	133/324 (41.0)	86/265 (32.5)	47/59 (79.7)	< 0.001
Female gender, %	161/324 (49.7)	137/265 (51.7)	24/59 (40.7)	0.126
BMI (kg/m^2^)	26.2 [23.2–29.0] [256]	26.2 [23.0–28.9] [214]	26.9 [23.2–31.7] [42]	0.337
CV risk factors				
Hypertension, %	173/324 (53.4)	147/265 (55.5)	26/59 (44.1)	0.112
Dyslipidemia, %	52/324 (16.0)	46/265 (17.4)	6/59 (10.2)	0.174
Comorbidity general				
Diabetes mellitus, %	35/324 (10.8)	31/265 (11.7)	4/59 (6.8)	0.271
Kidney failure (Crea. > 2.26 mg/dL)	8/324 (2.5)	7/265 (2.6)	1/59 (1.7)	1.000
Stroke, %	30/324 (9.3)	27/265 (10.2)	3/59 (5.1)	0.221
COPD, %	44/324 (13.6)	39/265 (14.7)	5/59 (8.5)	0.206
PAD, %	17/324 (5.2)	16/265 (6.0)	1/59 (1.7)	0.328
Comorbidity cardiac				
Atrial fibrillation, %	126/324 (38.9)	102/265 (38.5)	24/59 (40.7)	0.755
Coronary artery disease, %	45/324 (13.9)	41/265 (15.5)	4/59 (6.8)	0.081
Prior MI (≤90 days), %	4/324 (1.2)	3/265 (1.1)	1/59 (1.7)	0.554
Prior aortic valve replacement, %	15/324 (4.6)	11/265 (4.2)	4/59 (6.8)	0.489
Prior CABG, %	25/324 (7.7)	23/265 (8.7)	2/59 (3.4)	0.277
Prior pacemaker, %	13/324 (4.0)	11/265 (4.2)	2/59 (3.4)	1.000
NYHA class III/IV, %	273/324 (84.3)	225/265 (84.9)	48/59 (81.4)	0.498
CCS class III %^a^	16/324 (4.9)	12/265 (4.5)	4/59 (6.8)	0.505
Pulmonary hypertension, %	56/324 (17.3)	47/265 (17.7)	9/59 (15.3)	0.648
Emergency indication for surgery, %	31/324 (9.6)	23/265 (8.7)	8/59 (13.6)	0.249
Cardiac decompensation, %	112/324 (34.6)	95/265 (35.8)	17/59 (28.8)	0.304
Log EuroSCORE I (%)	7.2 [3.5–15.6] [322]	8.4 [3.6–17.5] [263]	4.5 [2.9–8.0] [59]	< 0.001

*Legend:* Values are patients applicable/patients with available information with percentage in brackets OR medians with IQR in brackets and available patient number in brackets; ^a^No patient presented with CCS class IV in this collective

*BMI* body mass index, *CV* cardiovascular, *CABG* coronary artery bypass graft, *CCS* Canadian Cardiovascular Society, *COPD* chronic obstructive pulmonary disease, *MI* myocardial infarction, *NYHA*,New York Heart Association, *PAD* peripheral artery disease, *SD* standard deviation

**Table 2:** MV pathologies and echocardiographic parameters

	Total *N* = 324	Biological MVR *N* = 265	Mechanical MVR *N* = 59	*p*-value
MV pathologies				0.712
Functional, %	13/324 (4.0)	10/265 (3.8)	3/59 (5.1)	
Degenerative, %	311/324 (96.0)	255/265 (96.2)	56/59 (94.9)	
Acute endocarditis, %	47/324 (14.5)	37/265 (14.0)	10/59 (16.9)	0.556
MV stenosis, %	93/324 (28.7)	71/265 (26.8)	22/59 (37.3)	0.107
Annulus dilatation, %	231/324 (71.3)	189/265 (71.3)	42/59 (71.2)	0.984
Annulus calcification, %	91/324 (28.1)	72/265 (27.2)	19/59 (32.2)	0.437
AML prolapse, %	78/324 (24.1)	69/265 (23.0)	17/59 (28.8)	0.346
AML flail, %	35/324 (10.8)	27/265 (10.2)	8/59 (13.6)	0.395
PML prolapse, %	119/324 (36.7)	99/265 (37.4)	20/59 (33.9)	0.618
PML flail, %	83/324 (25.6)	72/265 (27.2)	11/59 (18.6)	0.175
Chordae elongation, %	54/324 (16.7)	42/265 (15.8)	12/59 (20.3)	0.403
Restrictive leaflet, %	157/324 (48.5)	126/265 (47.5)	31/59 (52.5)	0.487
MV insuff. Grade ≥ II^a^, %	313/324 (96.6)	260/265 (98.1)	53/59 (89.8)	0.006
Echocardiographic parameters				
LVEF, %	56.0 [50.0–60.0] [324]	58.0 [50.0–60.0] [265]	55.0 [48.3–60.0] [59]	0.292
LVEDD (mm)	54.0 [49.0–59.0] [214]	54.0 [49.0–59.0] [200]	54.0 [50.0–57.0] [49]	0.950
LVESD (mm)	35.0 [31.0–40.5] [237]	35.0 [31.0–41.0] [191]	35.0 [32.0–40.0] [46]	0.444
Left atrium (mm)	55.0 ± 10.5 [251]	54.5 ± 10.2 [203]	57.2 ± 11.9 [48]	0.107
Right atrium (mm)	45.0 [38.0–53.0] [251]	45.0 [38.0–53.0] [203]	48.5 [39.3–54.5] [48]	0.318
Mitral opening (mm)	3.4 ± 1.8 [93]	3.4 ± 1.6 [79]	3.3 ± 2.6 [14]	0.966
PISA radius (mm)	1.0 [1.0–1.2] [56]	1.0 [1.0–1.2] [46]	1.0 [0.9–1.1] [10]	0.426
Vena contracta (mm)	6.0 [5.0–7.0] [90]	6.0 [4.0–7.0] [78]	6.0 [5.0–7.0] [12]	0.717

*Legend:* Values are patients applicable/patients with available information with percentage in brackets OR means ± SD with available patient numbers in brackets OR medians with IQR in brackets and available patient number in brackets; ^a^ patients with MR grade < II initially underwent mitral valve repair and, in cases where the result was not satisfactory, underwent mitral valve replacement

*AML* anterior mitral valve leaflet, *LVEDD* left ventricular end diastolic pressure, *LVEF* left ventricular ejection fraction, *LVESD* left ventricular end systolic pressure, *MV* mitral valve, *PISA* proximal isovelocity surface area, *PML* posterior mitral valve leaflet, *SD* standard deviation

### Procedural details and outcomes

Patients in the biological valve group received less minimally invasive MV surgery (MIC; 37.4% vs. 61.0%; *p* = 0.001) and more conventional sternotomy (CS) (Table 3). While the operating time was comparable between the two groups there was a shorter cardiopulmonary bypass (CPB) time in the biological valve group (*p* = 0.030). Hospital stay was lower in the biological valve group (Table 3) (*p* = 0.012). Noteworthy was a higher need for secondary MV repair (11.9 vs. 0.8%; *p* < 0.001) in the mechanical valve group.

**Table 3:** Procedural details

	Total *N* = 324	Biological MVR *N* = 265	Mechanical MVR *N* = 59	*p*-value
Operative approach				0.001
MIC, %	135/324 (41.7)	99/265 (37.4)	36/59 (61.0)	
CS, %	189/324 (58.3)	166/265 (62.6)	23/59 (39.0)	
MV size				0.652
25 mm, %	2/324 (0.6)	2/265 (0.8)	–	
26 mm, %	1/324 (0.3)	1/265 (0.4)	–	
27 mm, %	45/324 (13.9)	40/265 (15.1)	5/59 (8.5)	
29 mm, %	99/324 (30.6)	82/265 (30.9)	17/59 (28.8)	
31 mm, %	128/324 (39.5)	100/265 (37.7)	28/59 (47.5)	
33 mm, %	49/324 (15.1)	40/265 (15.1)	9/59 (15.3)	
Times				
Procedure time (min)	196.5 [166.3–240.0] [324]	197.0 [165.5–239.0] [265]	192.0 [170.0–258.0] [59]	0.539
CPB time (min)	117.0 [166.3–146.8] [324]	114.0 [94.0–145.0] [265]	126.0 [105.0–154.0] [59]	0.030
x-clamp time (min)	72.0 [59.0–92.8] [324]	71.0 [58.0–91.0] [265]	74.0 [62.0–99.0] [59]	0.235
Length of intubation (h)	12.0 [9.0–18.0] [324]	12.0 [9.0–18.0] [265]	11.0 [8.0–17.0] [59]	0.230
Length of ICU (h)	34.5 [22.0–75.8] [324]	34.0 [22.0–72.0] [265]	47.0 [23.0–108.0] [59]	0.095
Length of hospital stay (d)	12.0 [9.0–17.0] [324]	11.0 [9.0–17.0] [265]	14.0 [11.0–19.0] [59]	0.012
Concomitant procedures				
Cryo ablation, %	82/323 (25.4)	65/265 (24.5)	17/58 (29.3)	0.448
LAA closure, %	88/324 (27.2)	74/265 (27.9)	14/59 (23.7)	0.512
Tricuspid valve repair, %	63/323 (19.5)	53/264 (20.1)	10/59 (16.9)	0.584
PFO closure, %	13/324 (4.0)	10/265 (3.8)	3/59 (5.1)	0.712
ASD closure, %	6/324 (1.9)	5/265 (1.9)	1/59 (1.7)	1.000
Second MV repair, %	9/324 (2.8)	2/265 (0.8)	7/59 (11.9)	< 0.001
Conversion to CS^a^, %	9/137 (6.6)	9/101 (8.9)	0/36 (0)	0.112

*Legend:* Values are patients applicable/patients with available information with percentage in brackets OR medians with IQR in brackets and available patient number in brackets; ^a^Reasons for conversion were severe forms of trichterbrust, severely elevated diaphragm or severe adhesion of the right pleura, as well as severe intraoperative bleeding

*ASD* atrial septum defect, *CPB* cardiopulmonary bypass, *CS* conventional sternotomy, *ICU* intensive care unit, *LAA* left atrial appendage, *MIC* minimally invasive mitral valve surgery, *MV* mitral valve, *PFO* patent foramen ovale, *SD* standard deviation

Frequent procedure related complications after MVR were atrial fibrillation (21.0%) and pneumonia (9.9%) (Table 4). There were no statistically significant differences between groups. Immediate procedural mortality (1.9%) was only seen in patients undergoing biological valve replacement.

**Table 4:** Procedure-related complications

	Biological MVR *N* = 265	Mechanical MVR *N* = 59	Odds Ratio 95% CI	Adjusted Odds Ratio^a^ 95% CI
Wound infection, %	9/265 (3.4)	1/59 (1.7)	2.04 (0.25–16.41)	1.01 (0.11–9.77)
Pericardial tamponade, %	21/265 (7.9)	5/59 (8.5)	0.93 (0.34–2.58)	0.67 (0.22–2.05)
AV block grade III, %	22/265 (8.3)	5/59 (8.5)	0.98 (0.35–2.70)	0.57 (0.18–1.75)
Pneumonia, %	27/265 (10.2)	5/59 (8.5)	1.23 (0.45–3.33)	1.02 (0.35–2.97)
Pneumothorax, %	2/265 (0.8)	2/59 (3.4)	0.22 (0.03–1.57)	0.22 (0.02–1.98)
Pleural effusion, %	12/265 (4.5)	1/59 (1.7)	1.59 (0.19–13.44)	1.62 (0.18–14.45)
Atrial fibrillation, %	59/265 (22.3)	9/59 (15.3)	1.60 (0.74–3.44)	1.09 (0.47–2.51)
MVI ≥ II post OP, %	6/264 (2.3)	1/59 (1.7)	1.35 (0.16–11.42)	1.86 (0.20–17.31)
Immediate 72 h procedural mortality, %	6/265 (2.3)	0/59 (0)	n.a.	n.a.

*Legend:* Values are patients applicable/patients with available information with percentage in brackets

*AV* atrioventricular, *MVI* mitral valve insufficiency, *n.a.* not applicable

^a^Odds Ratios were calculated by logistic regression and adjusted for age, logistic EuroScore -I, CAD and MV insuff. Grade ≥ II (preOP)

### Echocardiography data

The mean post-operative diastolic MV gradient was 5.0 mmHg for all patients and patients receiving a biological valve, but 4.0 mmHg for patients receiving mechanical valves (*p* = 0.833). After a mean follow-up of 6.4 years, the difference between the groups became significant (*p* = 0.018). The recorded peak diastolic MV gradient was 13.0 mmHg in the biological valve group and 9.5 mmHg in the mechanical valve group (*p* = 0.117), after a mean follow-up of 5.7 years.

### Follow-up

At 30 days, 9.0% of all patients had died, 4.0% experienced stroke, 10.5% acute renal failure, 8.0% received a pacemaker and 14.2% had to undergo re-thoracotomy (Table 5). OR between groups were, however, not statistically different for either the unadjusted and adjusted analyses. Re-thoracotomies had the highest absolute difference of 9.5% (12.5% vs. 22.0%; adjusted OR 0.45, 95% CI 0.20–1.00; *p* = 0.050).

**Table 5:** 30-day Outcomes

	Biological MVR *N* = 265	Mechanical MVR *N* = 59	Odds Ratio 95% CI	Adjusted Odds Ratio^a^ 95% CI
Death, %	23/265 (8.7)	6/59 (10.2)	0.84 (0.33–2.16)	0.50 (0.17–1.52)
Cardiac death, %	15/265 (5.7)	4/59 (6.8)	0.88 (0.28–2.75)	0.66 (0.18–2.42)
Non-cardiac death, %	8/265 (3.0)	2/59 (3.4)	0.89 (0.18–4.29)	0.37 (0.06–2.32)
Stroke, %	11/265 (4.2)	2/59 (3.4)	1.23 (0.27–5.72)	0.47 (0.08–2.66)
Acute renal failure, %	27/265 (10.2)	7/59 (11.9)	0.85 (0.35–2.05)	0.45 (0.16–1.24)
Myocardial infarction, %	0/265 (0)	2/59 (3.4)	n.a.	n.a.
Pacemaker implantation, %	20/264 (7.6)	6/59 (10.2)	0.45 (0.16–1.29)	0.72 (0.28–1.89)
Re-thoracotomy, %	33/265 (12.5)	13/59 (22.0)	0.50 (0.25–1.03)	0.45 (0.20–1.00)

*Legend:* Values are patients applicable/patients with available information with percentage in brackets

*n.a.* not applicable

^a^Odds ratios were calculated by logistic regression and adjusted for age, logistic EuroScore-I, CAD and MV insuff. ≥ grade II (preOP)

The most frequent major complication was embolism, which occurred in 9.2% (19/229) of patients (9.2% [17/184] vs. 4.4% [2/45] in the biological and mechanical valve groups, respectively; *p* = 0.381) and mostly affected the brain. Bleeding complications occurred in 4.8% (11/229) of patients, but were significantly higher in the mechanical valve group (13.3% [6/45] vs. 2.7% [5/184], respectively; *p* = 0.009) (Additional file 1: Table S1). In Additional file 2: Figure S1 the occurrences of these two complications over time are shown as KM curves.

**Fig. 2:**
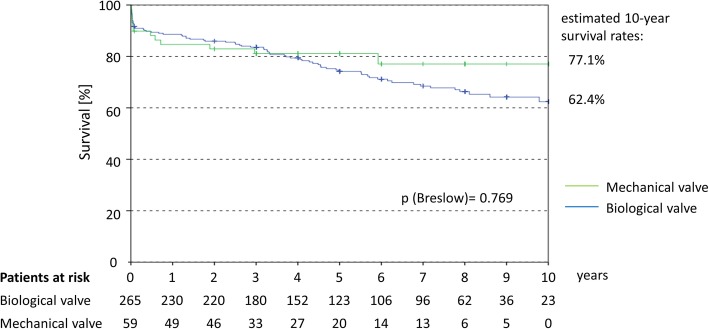
Kaplan–Meier curve for the long-term survival after MVR. *Legend:* HR calculated by COX regression was 1.264 (95% CI 0.687–2.325; *p* = 0.451) After adjustment for age, logistic EuroScore-I, CAD and MV regurgitation grade ≥ II (preOP) the HR was 0.833 (95% CI 0.430–1.615; *p* = 0.589) in favour for biological valves

In the overall follow-up period of up to 10 years, we found that during the first post-treatment years patients receiving a biological MV had a nominally better survival rate. 3 years post-treatment, this shifted nominally in favour of patients receiving mechanical valves. Survival rates were 62.4% for biological and 77.1% for mechanical valves at 10 years (*p* = 0.769). Differences were not statistically significant at any time point (Fig. 2). The HR was 1.264 (95% CI 0.687–2.325; *p* = 0.451). After adjustment for key baseline variables (age, CAD, MV regurgitation grade ≥ II, logistic EuroScore-I) no significant statistical difference between biological and mechanical valves was found (HR 0.833 (95% CI 0.430–1.615); *p* = 0.589).

## Discussion

This single centre study provides a contemporary picture of the safety and effectiveness of the two valve concepts. Event rates at 30 days may appear slightly high and we consider this the result of our clinic getting referrals of very complex cases that may result in longer procedural times and hazards. Furthermore our clinic is frequently chosen for re-interventions after failed procedures. Our study revealed a success rate of MVR that is quite comparable between valves, but confirmed that biological valves were used in older patients, while mechanical valves were used in younger patients and associated with an increased risk of bleeding.

Both types of valves have advantages and disadvantages, but also specific patient factors need to be taken into consideration when making the decision on which valve type to use [9, 10]. Historically, biological MVs were generally considered to have superior antithrombotic properties but lacked durability, while mechanical valves were thought to be more durable but were associated with thromboembolism and bleeding events [2, 5, 6, 9]. The data from our study confirm the observation that the use of anticoagulation along with mechanical valve implantation is associated with an increased risk of bleeding and that there is a potential survival benefit for patients receiving a biological MV.

Earlier studies report that mechanical valves were associated with increased durability [2, 5, 6]. The KM data confirm that at 10 years patient survival is higher with the mechanical valve than the biological valve (77.1% vs. 62.4%, respectively), despite survival being nominally higher with the biological valve within the first years of follow-up. Our Cox regression analysis revealed that after adjustment for key baseline variables there was no significant difference between the two valve types (HR 0.833; 95% CI 0.430–1.615; *p* = 0.589). Further information is needed to determine if the durability of the mechanical and biological valve affects patient survival because the biological valve recipients were 15 years older in our dataset. In addition, biological valves have been associated with an increased risk of re-operation and structural valve deterioration, which may start to occur at 3 years but, on the other hand, a durability of 12-plus years has also been reported [11–15].

Patient-specific factors, including age, surgical factors, comorbidities and patient preference, also influence the choice of valve type [9, 16]. With respect to the patient’s age, the general recommendations are that patients younger than 65 years should receive a mechanical valve because of their increased durability, while patients older than 65 years should be considered for a biological valve, as they are less likely to outlive the valve’s life expectancy [9, 17, 18]. Our study mirrors this approach, with the median age of patients in the mechanical valve group being 15 years lower. Comorbidities, such as atrial fibrillation, renal failure and diabetes, and surgical factors, such as the need for concurrent aortic root replacement, also affect valve selection [9, 19], although we did not observe any statistically significant difference in the rate of these comorbidities.

In the future, improvements in mechanical valve structure may lower the risk of thromboembolism thus potentially reducing the intensity of lifelong anticoagulation, which may result in a preference for these valve types [20]. Furthermore, newer oral anticoagulants may also make mechanical valves more attractive from both the patient’s perspective and from a medical standpoint [21].

### Limitations

Overall, 324 patients were included in this study. The majority of these study patients received a biological valve (*n* = 265) and only 59 patients received a mechanical valve. Furthermore, patients receiving mechanical valve were typically younger than those receiving biological valve. Finally, there is an evolution of surgical techniques over time. As such, we adjusted the outcomes for differences in baseline variables to overcome this limitation. Data on major complications and echo data, collected at the patient’s last follow-up visit, were not available for some patients as they only recently received their implant. The data, however, provide a useful insight into post-procedural major complications and echocardiographic data.

## Conclusions

Despite a significant passage of time since MVR was first performed, many of the findings remain the same – biological valves tend to be implanted in older patients while mechanical valves are preferred in younger patients but associated with a higher risk of bleeding. The estimated 10-year survival rate tended to be higher in patients receiving mechanical valves; but adjusted Cox regression analysis showed no significant difference between the two valve types. It appears, therefore, as if valve selection may not be as important for patient survival as prior data suggested.

## Additional files

Additional file 1: **Table S1.** Major complications (embolism/stroke and bleeding) and their localisation. *Legend:* Values are patients applicable/patients with available information with the percentage in brackets. MI, myocardial infarction. (DOCX 12 kb)

Additional file 2: **Figure S1.** Kaplan–Meier curves for embolism/stroke (A) and bleeding (B) complications. (TIF 1692 kb)

## Data Availability

The datasets used and analysed during the current study are available from the corresponding author on reasonable request.

## Abbreviations

adj: Adjusted
AHA: American Heart Association
AML: Anterior mitral valve leaflet
ASA: Acetylsalicylic acid
ASD: Atrial septal defect
BMI: Body mass index
CABG: Coronary artery bypass grafting
CAD: Coronary artery disease
CCS: Canadian Cardiovascular Society
COPD: Chronic obstructive pulmonary disease
CPB: Cardio-pulmonary bypass
CS: Conventional sternotomy
CV: Cardiovascular
EACTS: European Association for Cardio-Thoracic Surgery
ESC: European Society of Cardiology
HR: Hazard ratio
ICU: intensive care unit
IQR: Interquartile range
KM: Kaplan–Meier
LAA: Left atrial appendage
LV: Left ventricular
LVEDD: Left ventricular enddiastolic pressure
LVEF: Left ventricular ejection fraction
LVESD: Left ventricular endsystolic pressure
MI: Myocardial infarction
MV: Mitral valve
MVI: Mitral valve insufficiency
MVR: Mitral valve replacement
MVRep: Mitral valve repair
NYHA: New York Heart Association
OR: Odds ratio
PAD: Peripheal artery disease
PFO: Patent foramen ovale
PISA: Proximal isovelocity surface area
PML: Posterior mitral valve leaflet
SD: Standard deviation
TEE: Transoesophageal echocardiography
