# Supplementary material for: Long-term outcome after mitral valve replacement using biological versus mechanical valves
Source: J Cardiothorac Surg. 2019 Jun 28;14:120. doi: 10.1186/s13019-019-0943-6 (PMC6599286; doi:10.1186/s13019-019-0943-6)
Supplement: Supplementary file 1 — Table S1. Major complications (embolism/stroke and bleeding) and their localisation. Legend: Values are patients applicable/patients with available information with the percentage in brackets. MI, myocardial infarction. (DOCX 12 kb) [file 13019_2019_943_MOESM1_ESM.docx]

Table S1 Major complications (embolism/stroke and bleeding) and their localisation

|  | **Total**  **N = 324** | **Biological MVR**  **N = 265** | **Mechanical MVR**  **N = 59** | **p-value** |
| --- | --- | --- | --- | --- |
| **Embolism/stroke** | 19 / 229 (9.2) | 17 / 184 (9.2) | 2/45 (4.4) | 0.381 |
| Brain | 13 / 18 (72.2) | 13/16 (81.3) | 0 |  |
| Heart/ MI | 3 / 18 (16.7) | 1/16 (6.3) | 2/2 (100.0) |  |
| Others | 2 / 18 (11.1) | 2/16 (12.5) | 0 |  |
| **Bleeding** | 11 / 229 (4.8) | 5 / 184 (2.7) | 6 / 45 (13.3) | 0.009 |
| Brain | 3/11 (27.3) | 2/5 (40.0) | 1 / 6 (16.7) |  |
| Stomach | 1/11 (9.1) | 0 | 1/6 (16.7) |  |
| Urinary tract | 1/11 (9.1) | 0 | 1/6 (16.7) |  |
| Others | 6/11 (54.5) | 3/5 (60.0) | 3/5 (60.0) |  |

*Legend:* values are patients applicable / patients with available information with percent in brackets

MI, myocardial infarction
