# Supplementary figures and images for: Long-term outcome after mitral valve replacement using biological versus mechanical valves
Source: J Cardiothorac Surg. 2019 Jun 28;14:120. doi: 10.1186/s13019-019-0943-6 (PMC6599286; doi:10.1186/s13019-019-0943-6)

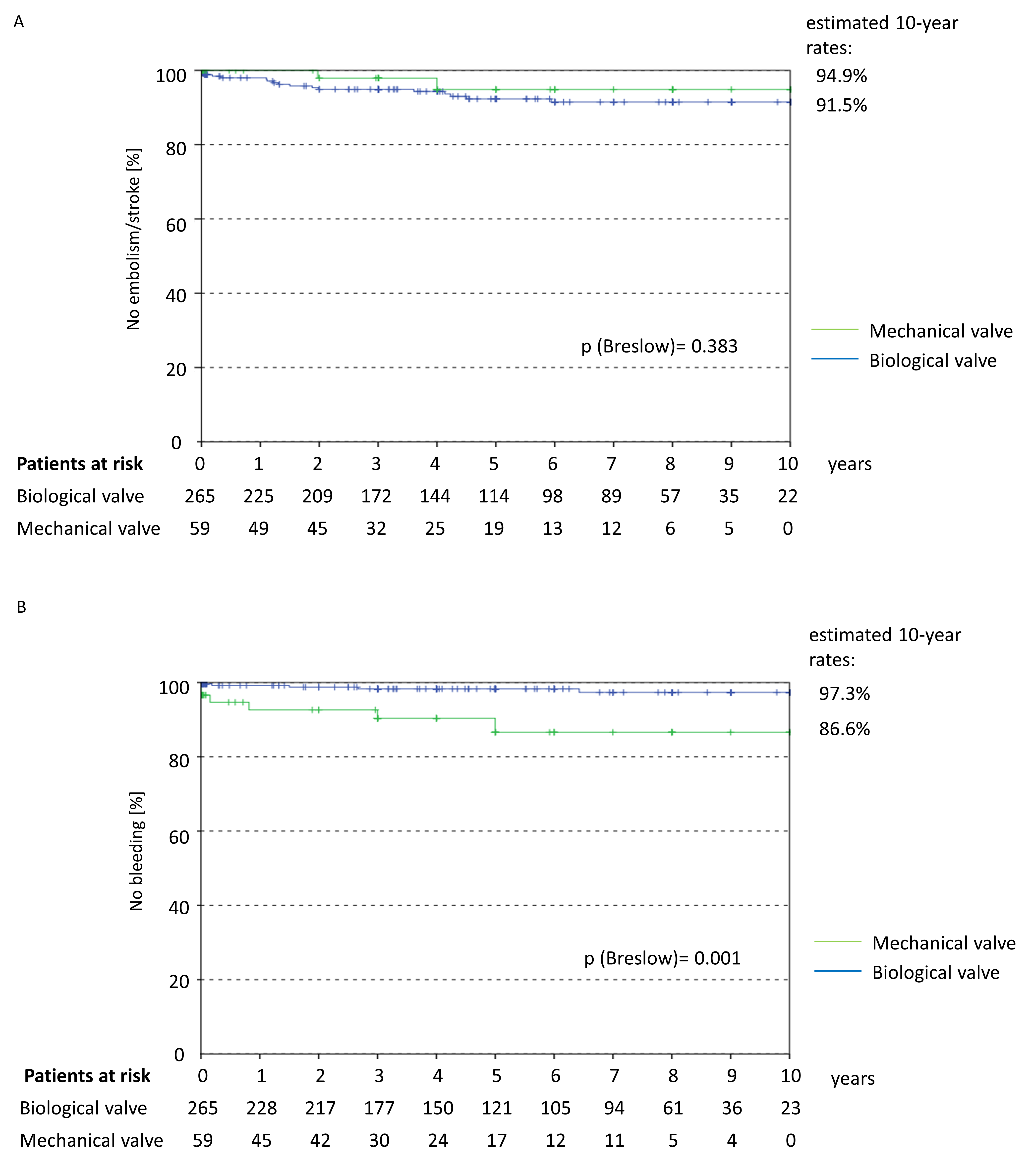

Supplement: Supplementary file 2 — Figure S1. Kaplan–Meier curves for embolism/stroke (A) and bleeding (B) complications. (TIF 1692 kb) [file 13019_2019_943_MOESM2_ESM.tif]
